# QTL mapping of seedling tolerance to exposure to low temperature in the maize IBM RIL population

**DOI:** 10.1371/journal.pone.0254437

**Published:** 2021-07-09

**Authors:** Raeann Goering, Siri Larsen, Jia Tan, James Whelan, Irina Makarevitch

**Affiliations:** Department of Biology, Hamline University, Saint Paul, Minnesota, United States of America; North Dakota State University, UNITED STATES

## Abstract

Maize is a cold sensitive crop that exhibits severe retardation of growth and development when exposed to cold spells during and right after germination, including the slowdown in development of new leaves and in formation of the photosynthetic apparatus. Improving cold tolerance in maize would allow early sowing to improve crop yield by prolonging a growing season and by decreasing the negative effects of summer drought, diseases, and pests. Two maize inbreds widely incorporated into American maize germplasm, B73 and Mo17, exhibit different levels of tolerance to low temperature exposure at seedling stage. In addition, thirty seven diverse inbred maize lines showed large variation for seedling response to low temperature exposure with lines with extremely low tolerance to seedling exposure to low temperatures falling into stiff stalk, non-stiff stalk, and tropical clades. We employed the maize intermated B73×Mo17 (IBM) recombinant inbred line population (IBM Syn4 RIL) to investigate the genetic architecture of cold stress tolerance at a young seedling stage and to identify quantitative trait loci (QTLs) controlling this variation. A panel of 97 recombinant inbred lines of IBM Syn4 were used to measure, and score based on several traits related to chlorophyll concentration, leaf color, and tissue damage. Our analysis resulted in detection of two QTLs with high additive impact, one on chromosome 1 (bin 1.02) and second on chromosome 5 (bin 5.05). Further investigation of the QTL regions using gene expression data provided a list of the candidate genes likely contributing to the variation in cold stress response. Among the genes located within QTL regions identified in this study and differentially expressed in response to low temperature exposure are the genes with putative functions related to auxin and gibberellin response, as well as general abiotic stress response, and genes coding for proteins with broad regulatory functions.

## Introduction

Maize (*Zea mays* L.) is the most widely distributed crop in the world with approximately 92 million acres planted in the United States every year [1, 2]. Since its worldwide spread, maize has become the most productive cereal crop and, therefore, is an extremely important, direct or indirect, source of food for humans. While in the United States, maize is used as a source for food and livestock feed, as well as for producing ethanol fuels, in many developing countries, it is grown by farmers and is used primarily as a food source. In Africa, for example, two-thirds of the produced corn is used for human consumption [3]. Thanks to historical improvements of cold tolerance, shortened growth cycle, and flowering adaptation to long days, maize, a tropical crop, is currently cultivated far outside of its original geographical region, in the environments where the temperatures regularly fall below optimal levels [4]. Only one-third of the total land mass is free of ice and 42% regularly experience temperatures below 20°C [5]. Maize is a cold sensitive crop that exhibits severe retardation of growth and development when exposed to cold spells during and right after germination, including the slowdown in development of new leaves [6] and in formation of the photosynthetic apparatus [4, 7]. Improving cold tolerance in maize would allow early sowing to improve crop yield by prolonging a growing season [8] and by decreasing the negative effects of summer drought, diseases, and pests [9].

Until recently, only a small number of genes and molecular pathways involved in maize response to cold stress were identified [10]. Genomics approaches during the last decades allowed to find many candidate cold-response genes, but revealed an important caveat. In transcriptomics analysis of plant response to cold stress, the tens of thousands of genes were found to change expression upon cold treatment with less than 500 reported in more than one study, suggesting that the maize response to cold stress is extremely variable, likely depending on the specific experimental conditions, plant stage, and plant material used [4]. Several quantitative trait locus (QTL) mapping of maize tolerance to low temperature exposure at different stages of plant development has been conducted on various maize populations, and dozens of QTLs related to different traits were identified [11–15] using such traits associated with tolerance to low temperature exposure, as chlorophyll fluorescence parameters, leaf greenness, leaf area, shoot dry weight, shoot nitrogen content, germination rate, and primary root length. These studies were conducted in different sets of maize lines, from biparental populations, such as the maize intermated B73xMo17 (IBM) population [13, 16], to multi-parent diversity panels, such as European diversity panels [17], and association panels [14, 18]. Most recently, a multi-parent advanced generations intercross population (MAGIC) identified hundreds of QTLs associated with various traits related to cold stress, primarily related to photosynthesis [15]. The large number of QTLs identified in these studies and moderate level of QTL overlapping between the studies strongly suggests a complex genetic architecture of tolerance to low temperature exposure, with numerous genes affecting various aspects of tolerance and acting at different stages of plant development. In addition, various maize lines likely carry different sets of alleles for these genes, suggesting that tolerance to low temperatures could be provided by multiple mechanisms that differ between maize lines. While multi-parent diversity and association panels provide many benefits for this analysis, the results of smaller QTL studies, especially when analyzed together with gene expression studies, could be highly beneficial for identifying alleles with strong effects.

Two maize inbreds widely incorporated into American maize germplasm, B73 and Mo17, exhibit different levels of tolerance to low temperature exposure at seedling stage. The maize intermated B73×Mo17 (IBM) recombinant inbred line population (IBM Syn4 RIL) was generated from B73 and Mo17 parental lines by four generations of intermating and eight generations of inbreeding [19], resulting in about 3.5 fold higher recombination compared to F_2_—derived recombinant inbred lines. In a recent QTL study, Mo17 showed lower germination rate and slower primary root growth rate compared to B73 under low temperature exposure and eight QTL regions related to low temperature exposure at the germination stage were identified (Hu et al., 2016). We were interested in investigating the response of B73 and Mo17 seedlings to low temperature exposure after germination.

In this study, a panel of 97 recombinant inbred lines of IBM Syn4 was used as a QTL mapping population to identify QTL regions controlling variation in tolerance to cold temperature exposure of maize seedlings as measured by the chlorophyll concentration, leaf color, and tissue damage. The genes located within identified QTL regions that are differentially expressed in response to low temperature exposure were identified and provide a pool of candidate genes for further investigating the molecular basis of cold tolerance in maize.

## Materials and methods

### Plant materials

Seed of 97 IBM Syn4 lines and their parents B73 and Mo17 were produced in summer 2010 by manual self-pollination of each plant at the University of Minnesota (Saint Paul campus) located at 44°59’35"N 93°10’28"W. This region has a humid continental climate and the average annual precipitation is 32”. After the seed ripened in the field, it was harvested and dried for fourteen days at ambient temperature and forced air. Seed was subsequently stored at 4°C in the cold rooms at the University of Minnesota and Hamline University. The average germination percentage of B73, Mo17, and IBM Syn4 population was 98%, 96%, and 94%, respectively.

### Cold tolerance experiments

B73, Mo17, and IBM Syn4 recombinant inbred lines (RILs) maize seedlings were grown in the growth chamber at 24°C in 1:1 mix of autoclaved field soil and MetroMix under 16 hour light: 8 hour dark conditions. For cold stress, 14-day old seedlings were incubated at 4°C for 8 hours in the dark and were allowed to recover for 24 hours at 24°C under normal day / night conditions. The humidity in the growth chamber was controlled at 30%. Three replicates of the cold stress exposure were conducted and ten seedlings per line were used for each replicate.

**Fig 1:**
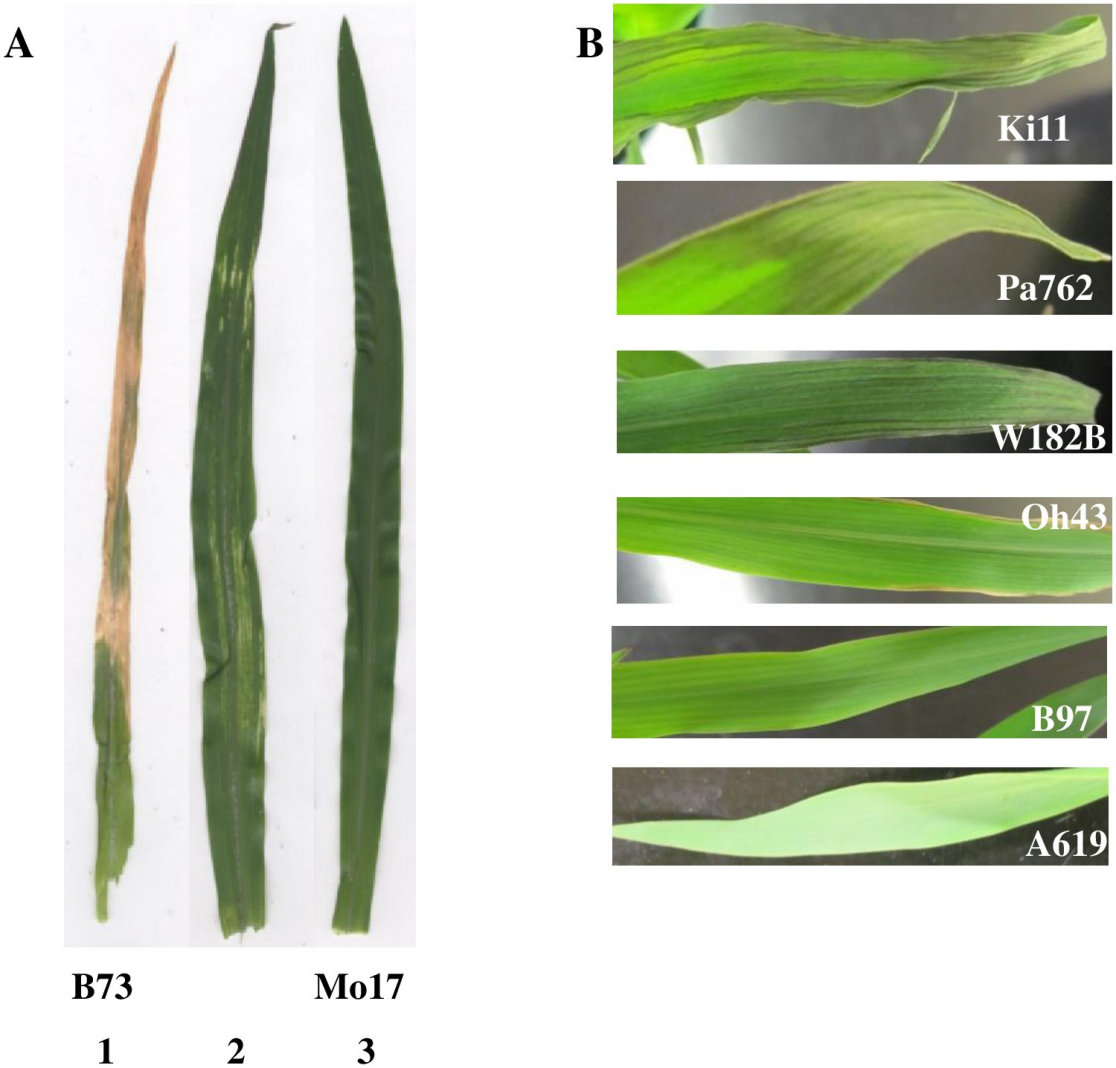
Effect of low temperature exposure of maize seedlings on leaf phenotype. A. The scale for visual scoring of tolerance to low temperature exposure was as follows: 1 –severe leaf damage, large proportion of dry / dead tissue, severe wilting; 2 –intermediate level of leaf damage, some discolored / dry tissue, primarily on the edges and tips of the leaf; 3 –minimal / no leaf damage, no discoloration, minimal wilting. B. Diverse maize lines vary in their response to low temperature exposure.

Plant tolerance to cold temperature was measured as the degree of leaf necrosis using several approaches. First, a score on the visual three-point scale was assigned to each plant with a score of 1 designating no leaf necrosis and the score of 3 designating significant leaf necrosis (Fig 1). Second, an image analysis of affected leaves was performed to quantitatively measure the degree of leaf necrosis. Briefly, the second and the third leaf of the seedlings were removed from the plants and scanned with a contrasting blue paper background. Image analysis was conducted in Mathematica (Wolfram Research, Inc., Version 10.4) using a function *Dominant Colors*. This function breaks an image into a specified number of color clusters, capturing any necrosis damage as dark green / gray, yellow or brown regions. The proportion of leaf area affected by the cold exposure was estimated as a proportion of pixels in the dark green / gray, yellow or brown regions of the leaf relative to the total area of the leaf. Finally, chlorophyll A and chlorophyll B were extracted by methanol from 500 ng of the leaf tip ground in liquid nitrogen [20]. Absorbance values at 663 nm and 645 nm were measured using Beckman Coulter DU530 UV/Vis spectrophotometer and used as the proxy measurement of concentrations of chlorophyll A and chlorophyll B, respectively. In addition, absorption values at 665 nm and 652 nm were converted to chlorophyll concentration using the following formulas from [21]: chlorophyll A [ug/ml] = 16.72 A_665_−9.16 A_652_; chlorophyll B [ug/ml] = 34.09 A_652_−15.28 A_665_. Chlorophyll A and chlorophyll B concentrations were predictably highly correlated with the measurements of absorbance at 663 nm and 645 nm; thus the absorbance values were used in the analysis as directly measured phenotypic data.

Data of 97 IBM lines were analyzed using analysis of variance (ANOVA) in SPSS 12.0 (SPSS Inc., Chicago). Significant differences of seedling tolerance to low temperature exposure between the two parental lines B73 and Mo17 were detected using Student’s t test (p < 0.01). Pearson correlation was used to estimate correlation between traits. The coefficients of variation (CV) for each trait were calculated as follows: CV = σ / μ * 100, where σ is the standard deviation and μ is the mean. Heritabilities (H^2^) for each trait were calculated, fitting the effects of genotype (G) and environment (E), as H^2^ = σ_G_^2^/ _(_σ_G_^2^ + σ_E_^2^/r)*100, where H^2^ is broad sense heritability, σ_G_^2^ is genotypic variance, σ_E_^2^ is error variance, and r is the number of replicates [22].

### DNA marker analysis and QTL analysis

DNA marker data used for QTL analysis were retrieved from the MaizeGDB website (http://www.maizegdb.org/data_center/qtl-data) and formatted to contain 97 IBM Syn4 lines used in this study. This data is based on the reference map constructed by [23] using IBM population [19] and is based on a set of over 1,850 markers. These markers include a combination of single copy RFLP (restriction length polymorphism fragments) probes and SSR (simple sequence repeats) primer sets span all ten maize chromosomes (S1 Table). In this map, marker information marked “A” corresponds to the genotype of B73, “B” to the genotype of Mo17, and “-” represents a missing value. The number of markers ranged from 138 on chromosome 8 to 346 on chromosome 1 (S2 Table). The total length of the IBM Syn4 linkage map was 7116 cM with an average interval size of 3.64 cM. Cold tolerance scores measured by all three methods were used in quantitative trait locus analysis using the qtl package in R [24]. Simple interval mapping (SIM) and composite interval mapping (CIM) were employed for QTL analysis [24, 25]. The logarithm of odds (LOD) threshold values for each trait were determined by 1,000 permutations at a p = 0.05 level. QTL positions were assigned underneath maximal LOD scores. Additive effects of the detected QTL and the percent of phenotypic variance explained by marker genotypes were estimated.

### RNA-seq data analysis

The genes differentially expressed in response to cold exposure in B73 and Mo17 lines were identified using RNA-Seq datasets described in [26, 27]. The genes located in the QTL regions were selected using the maize genome B73 RefGen_V3 5b+ gene models (maizegdb.org). The genes that (i) were expressed at the level of at least 1 read per million under cold or control conditions in either B73 or Mo17 genetic backgrounds and (ii) exhibited fold ratio of cold to control expression levels of over 2 or below 0.5 were considered differentially expressed.

## Results and discussion

### Phenotypic analysis of leaf chlorophyll concentration-related traits under normal and low temperature growth conditions

B73 and Mo17 maize inbred lines exhibited surprisingly different phenotypic changes in response to low temperature exposure (Fig 1A). While Mo17 plants showed only minimal wilting immediately after low temperature exposure, B73 plants exhibited severe wilting. After recovery period at optimal temperature, Mo17 plants were essentially indistinguishable from control plants grown under optimal temperature with only slight yellowing of the leaf edges shown in some plants. In stark contrast, B73 plants exhibited extreme yellowing of leaf tips and edges and severe leaf necrosis that frequently spread through the whole leaf and was especially profound in larger leaves. To quantify this phenotype, we employed several strategies (see Materials and methods for detailed description). First, the plants were scored visually on the 3-point scale. Second, the leaf photos were analyzed using computer image analysis to estimate the proportion of green leaf tissue. Finally, chlorophyll a and b were extracted from the leaves and their concentration relative to the leaf weight was estimated using absorbance values at 663 nm and 645 nm. Both parental lines B73 and Mo17 showed no leaf yellowing and necrosis when grown under optimal temperature conditions. These lines were significantly different from each other for all four measurements (Table 1, Student t-test p-value < 0.01).

**Table 1:** Leaf color related traits of B73, Mo17 and Syn4 RIL population and their heritabilities under low temperature conditions.

Traits (cold)	Parents		IBM RIL lines			
	B73	Mo17	Range	Mean	CV(%)	H^2^
Proportion of green tissue*	75.43±3.09	99.00±0.73	74.4–100.0	93.90	7.13	91.8%
Visual scoring of stress tolerance*	1±0	2.89±0.33	1.0–3.0	2.40	29.78	94.6%
Absorbance at 663nm*	0.46±0.04	0.88±0.05	0.37–0.93	0.73	23.85	88.5%
Absorbance at 645 nm*	0.35±0.07	0.57±0.04	0.29–0.60	0.47	17.52	82.5%

The traits designated with stars showed statistically significant differences between Mo17 and B73 parents (Student t-test).

We further screened 37 additional diverse inbred maize lines for seedling response to low temperature exposure and found that these lines vary widely in the degree of leaf tissue damage and wilting (Fig 1B, Table 2). Lines with extremely low tolerance to seedling exposure to low temperatures fell into stiff stalk, non-stiff stalk, and tropical clades [28], while lines with moderate and high tolerance were distributed across all groups.

**Table 2:** Diverse inbred lines show various degrees of seedling tolerance to low temperature exposure.

Diverse Inbred Lines	Level of tolerance	Diverse Inbred Lines	Level of tolerance
*Non-Stiff Stalk*		Stiff Stalk	
A619	High	A634	High
B97	Moderate	A680	Moderate
C49A	Moderate	B73	Low
CI44	High	H100	Low
CO125	Moderate	NC250	High
Ky21	High	NC294	High
M14	High	Sweet Corn	
M162W	Low	IA2132	High
Mo17	High/Moderate	IL101T	Moderate
Mo46	Low	IL14H	High
MoG	Low	Mixed	
Oh43	High/Moderate	B105	Moderate
Oh7B	High/Moderate	CO255	High
Pa762	Moderate	SD40	Moderate
R168	High	Popcorn	
R4	Moderate	HP301	Moderate
Va14	High	IDS69	High/Moderate
Va26	High	Sg1533	High
W182B	Low	Tropical	
W22	Low	Ki11	Low
W64A	Moderate	NC358	Moderate
WD	Moderate		

The average values of all four traits within IBM Syn4 lines were closer to those of Mo17 than B73 (Table 1). The four measurement approaches showed significant correlations (p-value < 0.01) between each other with correlation coefficients ranging from 0.75 for absorbance at 645 nm and visual scoring to 0.91 for proportion of green tissue estimated through image computational analysis and absorbance at 663 nm. Absorbance values at 645 nm, more directly correlating with chlorophyll b concentrations showed weaker (albeit significant) correlations to other traits, suggesting that image analysis and visual scoring of the leaves were more precise in estimating color changes associated with chlorophyll a concentration. The strong correlation between measured traits suggested that all approaches to document leaf color changes in response to low temperature exposure likely measure the same trait and are likely controlled by the same genetic factors. Coefficient of variation (CV) of the measures traits varied from 7% for computations image analysis of the leaf damage to 29% for visual scoring of the stress tolerance. Heritability (H^2^) values were high for all traits, with the lowest at 82% for absorbance at 645 nm and the highest at 94.6% for visual scoring of stress tolerance, suggesting that variation in these traits are primarily due to genetic differences between IBM lines and not due to environmental influences.

**Fig 2:**
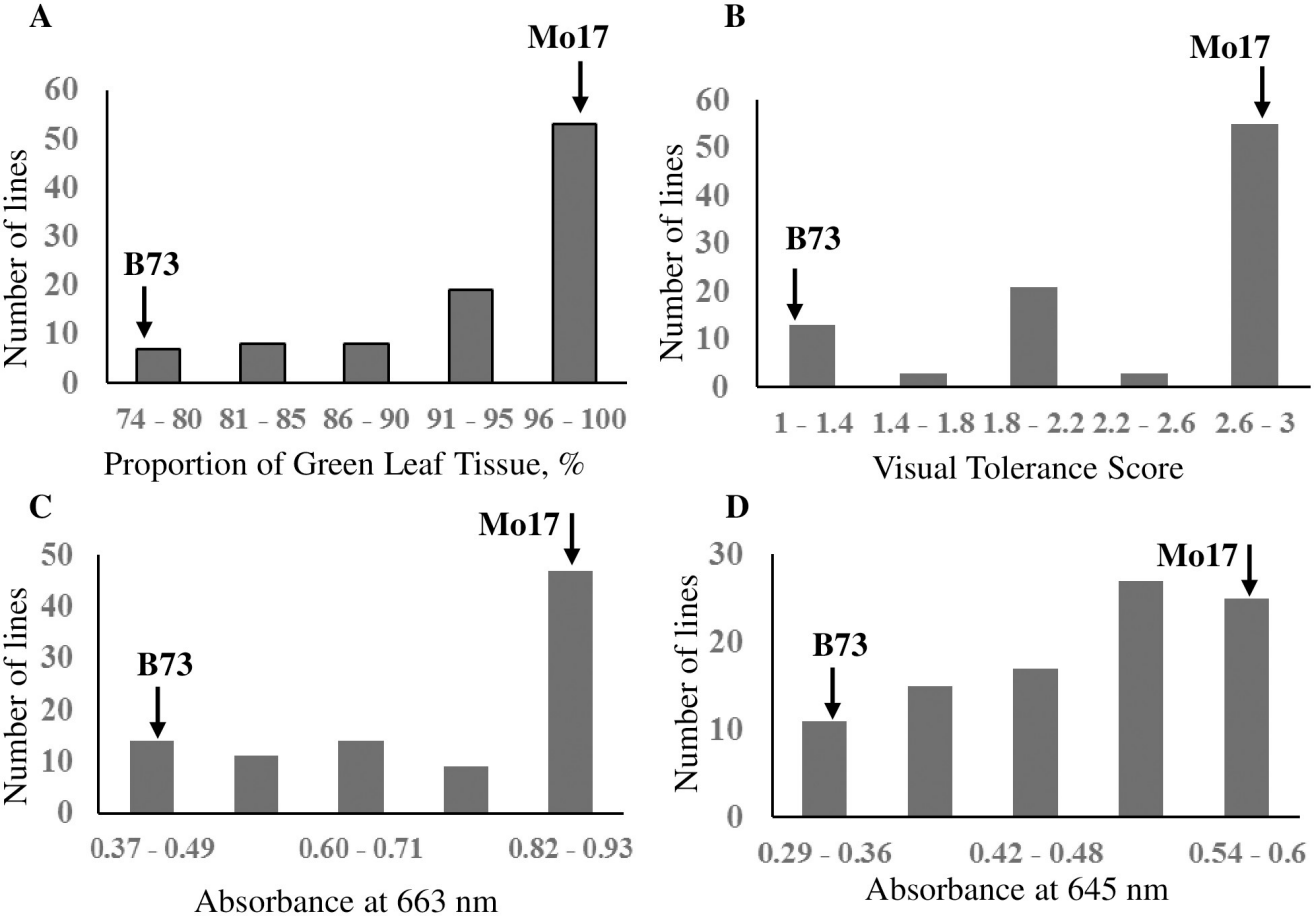
Frequency distributions of leaf color—Related traits following low temperature exposure in the IBM Syn4 population of RIL lines. A. Proportion of green leaf tissue following low temperature exposure; B. Visual cold tolerance score following low temperature exposure; C. Absorbance of methanol leaf extracts measured at 663 nm; D. Absorbance of methanol leaf extracts measured at 645 nm. Arrows show the relative position of B73 and Mo17 parents.

All measurements followed positively skewed distributions, with peak stress tolerance higher than the mean of the IBM Syn4 population (Fig 2). The number of lines showing B73-like and intermediate response were similar, while the majority of lines showed Mo17-like response to stress. Interestingly, Mo17 was not the highest scoring line, suggesting that B73 carries some genetic factors positively affecting plant tolerance to cold. The distribution ratio for the discrete visual scoring system was consistent with the additive effect of two dominant genetic factors having large effects on the trait.

### QTL analysis for leaf chlorophyll concentration-related traits under normal and low temperature growth conditions

**Fig 3:**
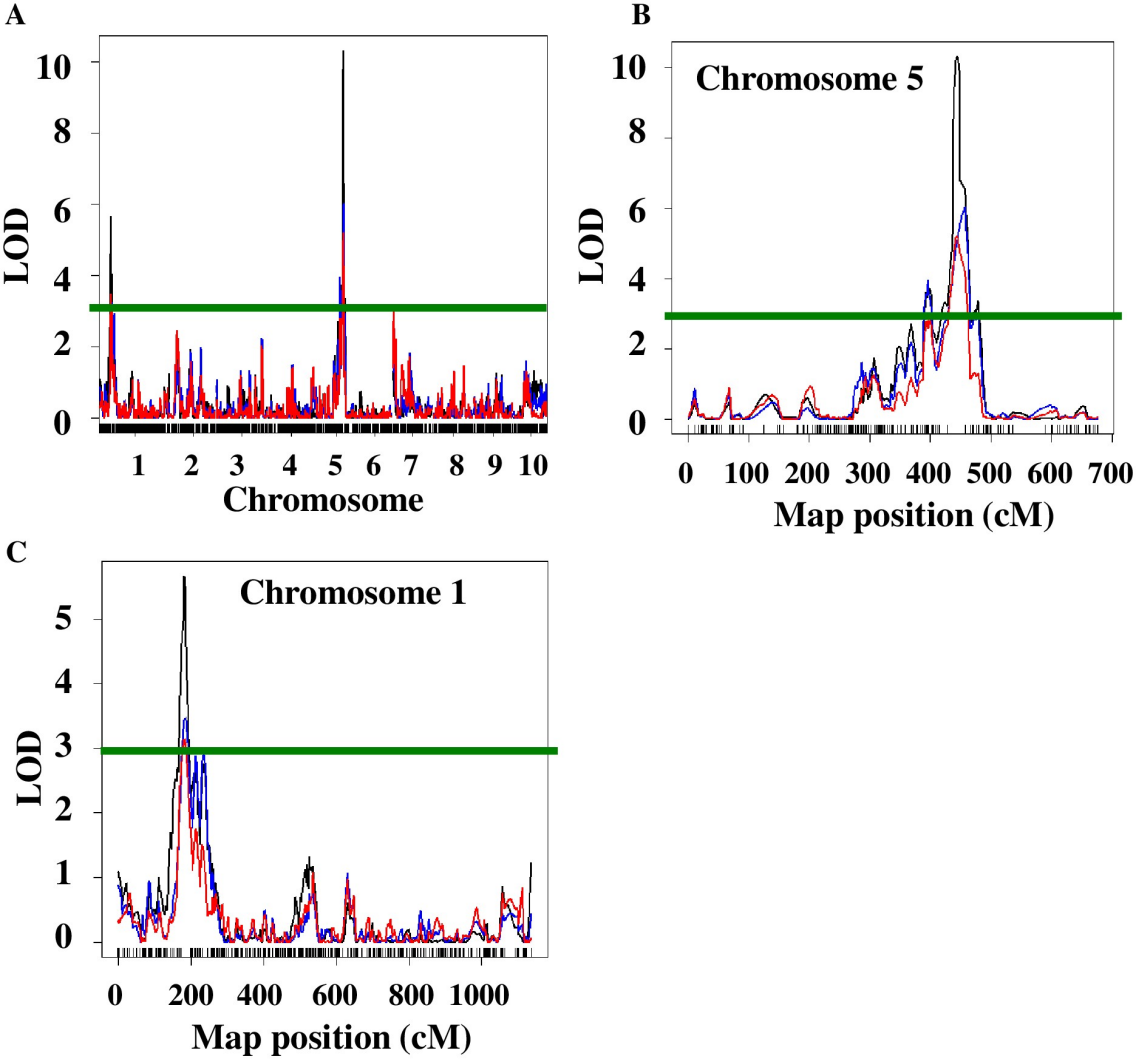
Profiles for QTLs for response to low temperature in maize seedlings in IBM Syn 4 population. LOD score of 3.0 (green line) was used as a cutoff to determine the statistical significance of discovered QTLs. The location of QTLs across the genome (A) or on chromosomes 5 (B) and 1 (C) are shown for various approaches used to measure plant response to cold temperature. The QTL profile determined using visual tolerance score is shown in black, using the proportion of green leaf tissue is shown in blue, while the QTL profile determined by absorbance at 645 nm is shown in red. The QTL profile determined using absorbance at 663 nm closely resembles the profile for visual tolerance score and is not shown.

QTLs were detected based on LOD thresholds after permutation tests using four phenotypic measurements (Fig 3). All four phenotypic measurements (visual cold stress tolerance score, proportion of green leaf tissue, and absorbance at 663 nm and 645 nm used to estimate the concentrations of chlorophyll a and b) identified two major QTLs (Table 3). Simple interval mapping (SIM) and composite interval mapping (CIM) were employed for QTL analysis [24, 25] and produced similar results. Only the results from SIM are shown. The LOD score for the QTL on chromosome 1 (QTL-1) varied from 3.4 to 6.8 depending on the phenotypic data set. The genetic region from B73 background for this QTL had a positive effect on stress tolerance, while the genetic region from Mo17 background for this QTL had a negative effect on stress tolerance. The QTL-1 explained 7.8–16.2% of the phenotypic variation. The LOD score for the QTL on chromosome 5 varied from 5.2 to 10.3 depending on the phenotypic data set. The genetic region from B73 background for this QTL had a negative effect on stress tolerance. The QTL-2 explained 14.8–27.9% of the phenotypic variation. Absorbance at 663 nm directly correlating to chlorophyll a concentration and visual tolerance score resulted in QTLs with larger LOD scores and a higher proportion of phenotypic variation explained. Among 97 IBM RILs, thirteen lines (MO051, MO054, MO068, MO088, MO090, MO105, MO123, MO126, MO134, MO146, MO157, MO171, MO183) have the highest phenotypic scores (average score of 3 in a visual test and an average score of 100% of green tissue). Twelve of these lines (except for MO088) carry a B allele of the QTL-1. Nine of these lines (except for MO054, MO126, MO146, MO171) carry an M allele of the QTL-2.

**Table 3:** QTLs detected for controlling leaf phenotype following low temperature exposure in the IBM Syn4 population.

QTL	Trait	Bin^a^	Peak position (cM)	Nearest marker	Left border (cM); 95% SI; flanking marker	Right border (cM); 95% SI; flanking marker	LOD Score^b^	P-value^c^	Additive Effect^d^	R^2^ (%)^e^
**QTL-1**	Visual tolerance score	1.02	181.7	cdo860a	174.7	186.7	5.7	3.1*10^−6^	-3.8	14.8
					gpm536c	uaz1				
	Proportion of green tissue	1.02	184.7	umc1711	174.7	238.7	3.4	3.3*10^−5^	-2.3	14.2
					gpm536c	IDP3914				
	Absorbance at 663 nm	1.02	182.7	IDP4750	172.7	190.7	6.8	4.9*10^−5^	-3.1	16.2
					sfp1					
	Absorbance at 645 nm	1.02	181.7	cdo860a	171.7	191.7	3.5	1.8*10^−4^	-2.8	7.8
					IDP7945	pco064372				
**QTL-2**	Visual tolerance score	5.05	445	gpm912a	442	448	10.3	8.7*10^−9^	5.2	23.3
					gpm912a	TIDP5693				
	Proportion of green tissue	5.05	449	umc1722	444	455	6.8	8.8*10^−8^	4.3	23.9
					myb86	lw3				
	Absorbance at 663 nm	5.05	448	IDP8645	443	454	9.6	3.7*10^−8^	5.0	27.9
					myb86	csu550				
	Absorbance at 645 nm	5.05	446	v12	441	453	5.2	2.8*10^−6^	4.1	14.8
					IDP8187	csu550				

^a^Chromosome bins, qtl and nearest marker positions are shown based on IBM 2008 Neighbors Map;

^b^LOD: Log10-likelihood value;

^c^p-value of the QTL fit is estimated for one QTL at a time and for the model (Q1 + Q2 + Q1*Q2);

^d^Additive effect: The negative value of additive effect indicates the allele from B73 is positive;

^e^R2: coefficient of determination, which represents the percentage of phenotypic variance explained by a putative QTL.

In the last several years numerous studies reported identifying multiple QTLs with various effects in different populations [11–15]. Specifics of the environmental conditions and genetic diversity of the lines utilized by the study likely impact the QTLs that are found by the studies, making direct comparisons of the QTLs found by the studies complicated. Both major QTLs found in our study were also detected by [15] as QTLs affecting various traits related to cold stress under growth chamber and field conditions, although it is difficult to assess if the QTLs in both studies are truly caused by the same variation due to different approaches of these studies. Several studies conducting the QTL analysis in the field and under the controlled growth chamber conditions underscored the difficulties in reproducing exact field conditions inside [13, 15]. The large fluctuations in climatic conditions was suggested as one of possible explanations for differences in QTLs identified in field and growth chamber experiments as additional parameters (for example, light intensity and water supply) are likely to change in the field conditions [29], while the climatic conditions are almost fixed in the growth chamber experiments. The recent study evaluating a large panel of maize inbreds via a genome wide association analysis revealed over 150 QTLs for emergence and traits related to early growth under cold stress [15]. Most of these QTLs had small effects and were specific for each environment. Interestingly, favorable alleles reported by [15] were found in the inbreds from different groups supporting our findings that favorable alleles for the two QTLs detected in our study came from two different parents: a favorable allele for the QTL-1 came from B73, while a favorable allele from the QTL-2 came from Mo17. Lines that carry both favorable alleles tend to show higher resistance to cold stress compared to Mo17, a parent with higher resistance to cold stress, consistent with additive effect of these two alleles. For example, all plants from lines carrying both favorable alleles received a maximum visual score (3±0), while Mo17 plants scored 2.89±0.33 on avearge. Analizing a larger number of plants from these lines could allow further quantifying the additive effect of favorable alleles for the QTLs 1 and 2. To further analyze the identified QTL regions, we found the genome location of the high confidence QTL regions identified by all phenotypic measurements and identified the genes located in these regions (Table 4). QTL– 1 spanned approximately 2.3 million base pairs on chromosome 1 and included 60 gene models (based on B73 RefGen_V3). QTL– 2 spanned over 3.4 million base pairs on chromosome 5 and included 69 gene models. Despite the relatively large physical length of the QTLs the number of genes in these regions is relatively small. To further characterize the genes located within QTL regions, we determined the transcription level of these genes under control and low temperature growth conditions in maize seedlings in B73 and Mo17 genetic backgrounds using the RNA-Seq data set from [27] since the growth conditions of the plants in the present study and the study reported in [27] were essentially identical. We hypothesized that the genes contributing to cold tolerance in seedling leaves would be expressed in leaves at this stage of plant development either under normal or low temperature growth conditions in either B73 or Mo17 genetic backgrounds. A total of 88 genes (42 within QTL—1 and 46 within QTL—5) satisfied this condition. We further suggested that the candidate genes contributing to cold tolerance would be differentially expressed in response to cold stress in the genetic background that provides a more tolerant allele (Mo17 for QTL– 5 or B73 for QTL -1), further reducing the number of possible candidate genes (Table 4). Among the genes located within QTL regions identified in this study and differentially expressed in response to low temperature exposure are the genes with putative functions related to auxin and gibberellin response, as well as general abiotic stress response, genes coding for proteins putatively involved in membrane transport, and genes putatively coding for broad regulatory proteins, such as a protein phosphatase, two protein kinases, and several transcription factors (S3 Table). Additional analysis is required to further differentiate among this list of potential candidate genes and to clone the genes within these QTLs that are contributing cold stress tolerance to maize seedlings. Interestingly, most of the candidate genes found in [15] were involved in metabolic processes and intracellular membrane-bounded organelles, suggesting that variation in intracellular membrane transport may be an important mechanism for cold tolerance.

**Table 4:** Physical Position and Gene Composition of Identified QTLs.

QTL	Chromosome	Left Border Position (nt)^a^	Right Border Position (nt)	Size (bp)	Genes^b^	Expressed Genes^c^	DE Genes in B73^d^	DE Genes in Mo17^d^
QTL—1	1	24,963,473	27,266,967	2,303,494	60	42	8	10
QTL—2	5	185,755,742	188,552,647	2,796,905	69	46	9	14

^a^Position of the QTLs on the physical map was determined using B73 RefGen_V4 version of the maize genome using the interval identified by all phenotypic measurements;

^b^Number of high confidence gene models based on B73 RefGen_V3 version of the maize genome;

^c^Genes expressed in seedlings under normal or low temperature conditions (RPM > 1) as determined using a data set from [27];

^d^Genes differentially expressed between normal and low temperature conditions (fold change of at least 2) as determined using a data set from [27].

Comparison of gene expression between two maize lines can reveal candidate genes responsible for phenotypic differences in cold tolerance when paired with a quantitative trait locus analysis. Natural genetic biological variation between the lines can also explain differences in how the lines respond to stress. In this analysis, 13 candidate genes potentially important for tolerance to early cold stress response were identified. These genes fall within cold tolerance QTL regions, are differentially expressed in response to cold, behave differently in cold responsive and tolerant lines and contain sequence variation between B73 and Mo17 likely resulting in altered gene function.

In conclusion, this study discovered two QTL regions controlling variation in tolerance to cold temperature exposure of maize seedlings as measured by the chlorophyll concentration, leaf color, and tissue damage, using a panel of 97 recombinant inbred lines derived from B73 and Mo17 inbred lines. Various quantitative and semi-quantitative approaches utilizing image analysis, visual scoring of seedling phenotype, as well as biochemical analysis of leaf chlorophyll concentration showed strong correlation between each other. The QTL regions on Chromosomes 1 and 5 explained 38% of phenotypic variation, when combined into a model with a Mo17 allele of the QTL on chromosome 5 providing tolerance to cold exposure and a B73 allele of the QTL on chromosome 1 providing tolerance to cold exposure. Further analysis is needed to clone specific genes and identify nucleotide variation explaining the large effect of these QTLs.

## Supporting information

S1 Table Phenotypic and genotypic data for Syn4 IBM RIL lines. (XLSX)

S2 Table Correlation between four measurements of leaf color in response to low temperature exposure. The values in the upper right portion of the table show correlation coefficients. All correlations were statistically significant (p<0.001), designated by *** in the lower left part of the table.
(XLSX)

S3 Table The location and potential function of potential candidate genes for cold QTL. ^a^Genes differentially expressed between normal and low temperature conditions (fold change of at least 2) as determined using a data set from [27].
(XLSX)
